# Cigarette, E-Cigarette and Waterpipe Use among Young Adults: Differential Cognitions about These Three Forms of Smoking

**DOI:** 10.3390/ijerph17113787

**Published:** 2020-05-27

**Authors:** Mark J.M. Sullman, Maria-Eugenia Gras, Antonios Kagialis, Ioulia Papageorgi, Sílvia Font-Mayolas

**Affiliations:** 1 Department of Social Sciences, University of Nicosia, Nicosia 2417, Cyprus; sullman.m@unic.ac.cy (M.J.M.S.); kaganthony@gmail.com (A.K.); papageorgi.i@unic.ac.cy (I.P.); 2 Department of Psychology, Universitat de Girona, 17004 Girona, Catalonia, Spain; eugenia.gras@udg.edu

**Keywords:** tobacco smoking, cigarettes, e-cigarettes, waterpipes, polytobacco, nicotine, young adults, cognitions, public health

## Abstract

Background: Polytobacco use is common among young adults. The purpose of the present study was to investigate a number of cognitions related to the use of three tobacco products (cigarettes, e-cigarettes and waterpipes) among young adults. Methods: Participants (*n* = 799, 59.4% women) aged 18–25 years old (M = 21.8, SD = 1.7) completed an online tobacco cognitions questionnaire. Results: For all three tobacco products, there was significantly more agreement with the cognition “I would smoke if my best friend offered” among tobacco users (used one or more tobacco products) than among non-users. For e-cigarettes and waterpipes, there was significantly more agreement with the cognition “It would be easy to quit these products” than was reported by non-users. Polytobacco users (three tobacco products) endorsed the cognitions scale (the six cognition items were combined to form a single cognitions scale for each tobacco product) significantly more than non-users for cigarettes and e-cigarettes. Furthermore, waterpipe users, polytobacco users, dual users and single users all endorsed the combined cognitions scales more strongly than non-users. Conclusions: Efforts to prevent polytobacco use should ensure that young adults have the necessary self-efficacy to resist peer pressure and provide them with clear information about the health risks associated with using alternative tobacco products.

## 1. Introduction

In most regions of the world, cigarette use continues to have a high prevalence among young adults [1,2,3]. Furthermore, the tobacco industry has responded to government regulations by promoting electronic nicotine delivery systems (e.g., e-cigarettes) and heated tobacco products (e.g., aerosols) as “cleaner” or “reduced risk” products [4].

E-cigarettes, and other alternative nicotine and tobacco products (ANTP), such as waterpipes (also known as hookah, shisha or narghile), have become increasingly attractive to young adults [5,6]. A substantial proportion of European Union citizens, especially young men, have tried waterpipes and systematic surveillance is required to monitor trends in their use [7]. According to the WHO Study Group on Tobacco Product Regulation [8] the literature on waterpipe use is relatively limited, but growing exponentially.

Polytobacco use, which is the concurrent use of alternative nicotine products and cigarettes, has been found to be more prevalent among young adults (18–25 years) than in older adults (26–34 years) [9,10,11]. Polytobacco use has also been found to be a predictor of greater cigarette use over time. Moreover, polytobacco use leads to an increased risk of nicotine addiction and other health risks among young adults, such as a higher probability of substance use disorders for alcohol and marijuana [12].

Previous studies have investigated the relationship that tobacco product use has with tobacco-related perceptions and attitudes among young adults [13]. Individuals with more positive attitudes towards waterpipes and e-cigarettes have been found to use these products more frequently [14]. Moreover, positive attitudes towards these tobacco products (cigarettes, cigars, chewing, hookah and e-cigarettes) has also been found to be associated with e-cigarette use [15]. Furthermore, the number of tobacco products tried has also been found to be predictive of perceived health risk, in that those who have tried more tobacco products report a lower perceived risk from their use [16,17].

More research into the cognitions related to polytobacco use among young adults is needed in order to better understand this increasing phenomenon and to help develop effective prevention programs, especially for emerging ANTPs, such as waterpipes. The present study set out to investigate a number of cognitions surrounding the use of cigarettes, e-cigarettes and waterpipes among young adults.

## 2. Materials and Methods

### 2.1. Participants and Procedure

The study design was based upon previous polytobacco research [14]. A convenience sample of 799 undergraduate students completed an online survey hosted by Google forms (Supplementary File S1 Questionnaire). The survey contained 24 closed questions, which were based on previous research [14,17], and was open from December 2019 to February 2020. Participation was voluntary without any form of inducement. Participants were recruited by emailing students studying psychology at a university in Turkey and another in Cyprus. These students were also asked to forward the survey onto other university students studying in their country. In both cases, English was the language of instruction and a minimum IELTS score of 6.5 was required. Participants self-selected into the study and provided informed consent. The inclusion criteria were that they were aged between 18 and 25 years old and were able to read and understand English [18]. Ethics permission was received from the Human Research Ethics Committee at Middle East Technical University—Northern Cyprus Campus and the Social Science Ethics Research Board at the University of Nicosia (SSERB 0054).

### 2.2. Measures

#### 2.2.1. Demographics

Participants reported their sex, age and ethnicity.

#### 2.2.2. Tobacco Use

Current tobacco use was assessed using the question “How frequently have you smoked cigarettes/e-cigarettes/waterpipes during the past 30 days?”. For each tobacco product, the response options were: “never”, “occasionally”, “once a week”, “more than once a week, but not every day”, and “every day”. 

Participants who reported “never” using tobacco were classified as “non-users” and those who answered “occasionally”, “once a week”, “more than a week, but not every day” or “every day” were classified as “current users”. Furthermore, those who reported the use of one tobacco product were classified as “single product users”, those who reported the use of two products were classified as “dual users” and those who reported using three products were classified as “poly users” [17].

#### 2.2.3. Tobacco Use Cognitions

The cognitions associated with tobacco use were based upon previous research in this area [19]. The questions were “I would smoke if my best friend offered”, “young people who use these products have more friends”, “the product makes young people look cool”, “the product makes young people feel more comfortable”, “the product helps to relieve stress”, and “it would be easy to quit these products”. Participants were asked to report how likely they were to endorse each cognition for each tobacco product (cigarette, e-cigarette and waterpipe). The response options for each of the six cognitions, and for each of the three tobacco products, were: “definitely yes”, “probably yes”, “probably no” and “definitely no”.

In order to construct the tobacco use cognitions scales (TUCS), the cognitions were dichotomized into “would not endorse” (definitely no and probably no), rated 0, or “would endorse” (definitely yes and probably yes), rated 1 [19]. Following this, we added the scores of the six items so that the scales created for each tobacco product ranged from 0 (no endorsement of any cognition) to 6 (endorsed all 6 cognitions) [15]. The Cronbach’s alphas for the scales were 0.70 for cigarettes, 0.73 for e-cigarettes and 0.80 for waterpipes.

### 2.3. Statistical Analysis

Chi-square tests were used to compare percentages, while t-tests and ANOVAs were used to compare means. Effect sizes were computed using the phi coefficient, Cohen’s d and eta squared. All analyses were performed using SPSS v23 manufactured by IBM Corp. (Armonk, NY, USA).

## 3. Results

### 3.1. Participants Demographics

The majority of the participants were female (*n* = 475; 59.4%) and had a mean age of 21.8 (SD = 1.7). Most participants identified themselves as Turkish (60.6%), followed by Greek, Greek Cypriot or Turkish Cypriot (21.8%), other European (5.3%) and other ethnicity (12.4%).

Among those who had used tobacco in the last 30 days, women were less likely than men to report cigarette (52.4% vs. 63.6%; χ^2^_(2)_= 9.8; *p* = 0.002; Φ = 0.11) and e-cigarette use (25.1% vs. 36.4%; χ^2^_(2)_ = 11.9; *p* = 0.001; Φ = 0.12), but no significant differences were found for waterpipe use (46.3% vs. 48.5%; χ^2^_(2)_ = 4; *p* = 0.55). With regard to age and ethnicity, current cigarette users were older (mean 22.0 vs. 21.6; t = −2.8; *p* = 0.004; d = 21) and were more likely to be Turkish (Turkish = 61.8%/Greek = 47.7%/Other European: 52.4%/Other Ethnicity: 51.5%, χ^2^_(2)_= 2.2; *p* = 0.007; Φ = 0.12), than non-users, but no differences were observed for e-cigarette or waterpipe use. 

### 3.2. Tobacco Use

The majority of participants were tobacco products users (*n* = 551; 72.7%), with cigarette use in the past 30 days being the most common (56.9%), followed by waterpipes (47.2%) and e-cigarettes (29.7%). Among current users, 30% were classified as single users, 22.7% as dual users and 19.1% were classified as polytobacco users.

### 3.3. Tobacco Use Cognitions

For all three products (cigarettes, e-cigarettes and waterpipes), there was significantly more agreement with the cognition “I would smoke if my best friend offered” among current users (used one or more tobacco product) than among non-users (Table 1). All three types of tobacco users (single, dual and poly users) endorsed the cognition “It would be easy to quit these products” significantly more than non-users, but only for e-cigarettes and waterpipes. There was no significant difference for cigarettes.

Current cigarette users (single, dual and poly users) endorsed the cognition “the product helps people relieve stress” significantly more than non-users. Furthermore, dual or poly users endorsed the cognition “the product makes young people feel more comfortable” significantly more than non-users. In addition, single and poly waterpipe users endorsed the cognition “The product helps people relieve stress” significantly more than non-users and dual users.

There were statistically significant differences in the tobacco use cognitions scales (TUCS) by polytobacco use status (Table 2). For cigarettes and e-cigarettes, poly users endorsed these cognitions significantly more than non-users. Moreover, for waterpipes, poly users, dual users and single users reported more positive cognitions than non-users.

## 4. Discussion

The present research found that the cognition “I would smoke if my best friend offered” was strongly associated with tobacco product use (cigarette, e-cigarette and waterpipe) and polytobacco use among young adults, in agreement with previous research on adolescents [19]. Moreover, we found that the endorsement of this cognition increased in accordance with the number of tobacco products used. These results are consistent with social learning theory [20] and provide further evidence of the important role social influence plays in smoking, as previously reported for ANTP use among adolescents [21,22] and for polytobacco use in young adults [15,23]. This pattern of findings appears to indicate that young adults are just as susceptible to social pressure as adolescents. Unfortunately, we do not know whether these young adults received any form of education or training to combat social influence, but the majority of these participants were not able to say no to any of the tobacco products when their friends were offering. Therefore, it is clearly important to improve self-efficacy to say no during early adolescence. However, given the fact that all our participants were university students, it seems that this type of intervention would be useful for young adults.

For e-cigarette and waterpipe use, the cognition “It would be easy to quit” was related to use of these products and to polytobacco use, as previously found among adolescents [19]. In contrast, this cognition was not significantly related to cigarette use. In support of these findings, previous research has found that young adults, who were single and dual tobacco product users, rated e-cigarettes and waterpipes as being less harmful than non-users [17]. Perhaps this pattern of results may be due to campaigns from the tobacco industry to promote ANTPs as being safer than regular cigarettes [4]. Therefore, tobacco counter-marketing campaigns are needed to target young adults [24]. Furthermore, interventions should not focus solely on traditional cigarettes, but must also include other forms of nicotine delivery to help the public understand that, irrespective of the delivery process, nicotine use has well known negative health consequences [25]. 

The cognitions “The product helps people relieve stress” and “The product makes young people feel more comfortable” were both associated with traditional cigarette use. Moreover, relieving stress was also associated with single and poly users for waterpipe use. Previous research has found that questions measuring affect regulation, such as boredom reduction, were predictive of polytobacco use among young adults [18]. Furthermore, previous research has also found that cognitions about affect regulation were associated with e-cigarette and waterpipe use among young adults [14] and waterpipe use among adolescents [19]. Therefore, it appears that young adults who use traditional cigarettes and ATNPs may be seeking a way to mitigate negative emotional affect. Future research is needed into the differential role that affect regulation has for cigarette users and ATNP users, in which type(s) of situations they use this behaviour and which alternative behaviours they could engage in as an alternative to drug use.

Consistent with previous studies (Lee et al., 2018) [15], our findings showed that polytobacco users (cigarettes, e-cigarettes and waterpipe) reported more positive cognitions about cigarettes and e-cigarettes than non-users. Significant differences were not found for single and dual tobacco users. Therefore, as other authors have proposed, it appears that the use of more tobacco products is associated with more positive cognitions, a reduction in the perceived risk of tobacco use, and the sustained use of these products [17,26]. Moreover, for waterpipe use, more positive cognitions were found for all types of currents users (single, dual and poly users), than were reported by non-users. These results seem to indicate that waterpipes are positively perceived by tobacco and ATNP users, which could be related to the increasingly widespread use of waterpipes around the world. 

Previous research has considered young adults to be particularly inclined to use waterpipes and has reported the need for public health messages to prevent the initiation and progression of waterpipe use [27,28,29]. Further research is needed into the relationship between waterpipe use and smoking multiple products, including the role of waterpipe use in the initiation or maintenance of other forms of tobacco use [30].

The present study has several limitations. Firstly, as this was a cross-sectional survey, only associations between the variables can be reported. Secondly, only two alternative nicotine products were investigated (e-cigarettes and waterpipes), so it is not possible to make conclusions about other alternative nicotine products. The study also used a convenience sample of university students that may not be representative of all young adults in Turkey and Cyprus. Furthermore, all measures relied on self-reported behaviour, which may be vulnerable to a number of potential biases, such as social desirability bias. 

The present study also has a number of strengths. Firstly, the present study was conducted in a population that differs substantially from those normally used to research health-related behaviours. Furthermore, the present study provides new information about the cognitions associated with polytobacco use in this novel population. In addition, the present study provided more information about the cognitions associated with waterpipe use. Another innovative element of the present study was the creation of a tobacco use cognitions scale. 

## 5. Conclusions

Interventions to prevent polytobacco use should focus on ensuring that young adults have the necessary self-efficacy to resist peer pressure. These interventions must also provide clear and complete information about the health risks associated with these alternative tobacco products. Future prevention campaigns must also be designed to take into consideration these cognitions about waterpipes, cigarettes and e-cigarettes. Future research is also needed to understand the transitions between products, as well as the cognitions associated with the maintenance of tobacco product use.

## Figures and Tables

**Table 1:** Cognitions (percentage of answers “probably yes” or “definitely yes”) by polytobacco status.

Substance	Cognition	Nonusers	Single Users	Dual Users	Poly Users	X^2^_(3)_ (*p*)	Φ
Regular cigarettes	I would smoke if my best friend offered	38.9 ^a^	56.0 ^b^	61.5 ^b^	62.1 ^b^	28.8 (<0.001)	0.19
	Young people who use these products have more friends	48.4	56.8	57.1	55.6	4.4 (0.22)	--
	The product makes young people look cool	42.1	41.1	41.9	39.9	0.4 (0.95)	--
	The product makes young people feel more comfortable	42.1 ^a^	50.2 ^a,b^	54.9 ^b^	60.1 ^b^	13.2 (0.04)	0.13
	The product helps people relieve stress	44.8 ^a^	58.5 ^b^	60.4 ^b^	62.1 ^b^	15.6 (0.001)	0.14
	It would be easy to quit these products	35.3	34.9	37.4	41.2	1.9 (0.60)	--
E-cigarettes	I would smoke if my best friend offered	32.6 ^a^	49.6 ^b^	52.7 ^b^	58.8 ^b^	30.0 (< 0.001)	0.19
	Young people who use these products have more friends	45.2	52.1	50.5	51.0	2.4 (0.49)	--
	The product makes young people look cool	42.5	36.4	36.8	40.5	2.4 (0.49)	--
	The product makes young people feel more comfortable	42.5	41.7	47.8	52.3	5.4 (0.15)	--
	The product helps people relieve stress	45.2	45.9	47.8	58.2	7.4 (0.06)	--
	It would be easy to quit these products	38 ^a^	49.2 ^b^	52.2 ^b^	49.7 ^b^	10.0 (0.02)	0.11
Waterpipe	I would smoke if my best friend offered	37.1 ^a^	49.0 ^b^	48.6 ^b^	66.7 ^c^	31.6 (< 0.001)	0.20
	Young people who use these products have more friends	41.6	43.5	39.3	44.7	1.1 (0.77)	--
	The product makes young people look cool	37.4	38.2	34.3	43.9	3.0 (0.39)	--
	The product makes young people feel more comfortable	41.6	46.9	46.9	53.3	4.9 (0.18)	--
	The product helps people relieve stress	45.2 ^a^	54.8 ^b^	42.5 ^a^	56.2 ^b^	10.6 (0.01)	0.12
	It would be easy to quit these products	39.4 ^a^	56.4 ^b^	49.2 ^b^	51.6 ^b^	13.9 (0.003)	0.13

Note: χ^2^—Chi-square value; *p*—significance; Φ—effect size coefficient phi; Significant pairwise comparisons are depicted by non-matching superscript letters, by row.

**Table 2:** Means and standard deviations for the tobacco use cognitions scales (TUCS) by polytobacco status.

TUC scales	Nonusers	Single Users	Dual Users	Poly Users	F (*p*)	η^2^
Regular cigarettes	2.4 (2.0) ^a^	2.9 (1.8) ^a,b^	2.5 (1.7) ^a,b^	3.0 (1.7) ^b^	4.8 (0.003)	19
E-cigarettes	2.5 (1.9) ^a^	2.8 (1.6) ^a,b^	2.9 (1.6) ^a,b^	3.1 (1.6) ^b^	4.7 (0.003)	17
Waterpipes	2.5 (1.9) ^a^	3.0 (1.6) ^b^	3.1 (1.4) ^b^	3.2 (1.5) ^b^	7.4 (<0.001)	27

Note: F—Snedecor F value; *p*—significance; η^2^—effect size coefficient eta square; Significant pairwise comparisons are depicted by non-matching superscript letters by row.

## Supplementary Materials

The following are available online at https://www.mdpi.com/1660-4601/17/11/3787/s1, Supplementary File S1 Questionnaire.
