# Supplementary material for: Cigarette, E-Cigarette and Waterpipe Use among Young Adults: Differential Cognitions about These Three Forms of Smoking
Source: Int J Environ Res Public Health. 2020 May 27;17(11):3787. doi: 10.3390/ijerph17113787 (PMC7312077; doi:10.3390/ijerph17113787)
Supplement: Supplementary file 1 [file ijerph-17-03787-s001.pdf]

**Supplementary File S1: Questionnaire (Adapted from Barnett & Livingston [19])**

How frequently have you smoked regular cigarettes during the past 30 days?

□ Never
□ Occasionally
□ Once a week
□More than once a week, but not everyday
□ Everyday

How frequently have you smoked e-cigarettes during the past 30 days?

□ Never
□ Occasionally
□ Once a week
□ More than once a week, but not everyday
□ Everyday

How frequently have you smoked from a waterpipe during the past 30 days?

□ Never
□ Occasionally
□ Once a week
□ More than once a week, but not everyday
□ Everyday

The following questions ONLY relate to CIGARETTE use. Please indicate how strongly you endorse (agree with) the following statements (in relation to cigarettes). How strongly do you endorsed the following statements for traditional cigarettes?

I would smoke if my best friend offered.

□ Definitely yes
□ Probably yes
□ Probably no
□ Definitely no

Young people who use these products have more friends.

□ Definitely yes
□ Probably yes
□ Probably no
□ Definitely no

The product makes young people look cool.

□Definitely yes
□ Probably yes
□ Probably no
□ Definitely no

The product makes young people feel more comfortable.

□ Definitely yes
□ Probably yes
□ Probably no
□ Definitely no

The product helps relieve people's stress.

□ Definitely yes
□ Probably yes
□ Probably no
□ Definitely no

It would be easy to quit these products.

□ Definitely yes
□ Probably yes
□ Probably no
□ Definitely no

The following questions ONLY relate to e-cigarette use. Please indicate how strongly you endorse (agree with) the following statements (in relation to e-cigarette use).

I would smoke if my best friend offered.

□ Definitely yes
□ Probably yes
□ Probably no
□ Definitely no

Young people who use these products have more friends.

□ Definitely yes
□ Probably yes
□ Probably no
□ Definitely no

The product makes young people look cool.

□ Definitely yes
□ Probably yes
□ Probably no
□ Definitely no

The product makes young people feel more comfortable.

□ Definitely yes
□ Probably yes
□ Probably no
□ Definitely no

The product helps relieve people's stress.

□ Definitely yes
□ Probably yes
□ Probably no
□ Definitely no

It would be easy to quit these products.

□ Definitely yes
□ Probably yes
□ Probably no
□ Definitely no

The following questions ONLY relate to SHISHA or water pipe use. Please indicate how strongly you endorse (agree with) the following statements (in relation to Shisha or water pipe use).

I would smoke if my best friend offered.

□ Definitely yes
□ Probably yes
□ Probably no
□ Definitely no

Young people who use these products have more friends.

□ Definitely yes
□ Probably yes
□ Probably no
□ Definitely no

The product makes young people look cool.

□ Definitely yes
□ Probably yes
□ Probably no
□ Definitely no

The product makes young people feel more comfortable.

□ Definitely yes
□ Probably yes
□ Probably no
□ Definitely no

The product helps relieve people's stress.

□ Definitely yes
□ Probably yes
□ Probably no
□ Definitely no

It would be easy to quit these products.

□ Definitely yes
□ Probably yes
□ Probably no
□ Definitely no
